# Overcoming Biological Barriers and Drug Resistance Through Next-Generation Nanotherapeutic Delivery in Gastric Cancer

**DOI:** 10.3390/cells15100850

**Published:** 2026-05-07

**Authors:** Md Ataur Rahman, Maroua Jalouli, Abdel Halim Harrath, Jinwon Choi, Min Choi, Hyo Jeong Kim, Sohyun Park, Bum-Sang Shim, Amama Rani, Bonglee Kim

**Affiliations:** 1 Department of Oncology, Karmanos Cancer Institute, Wayne State University, Detroit, MI 48201, USA; ataur1981rahman@hotmail.com; 2 Department of Biology, College of Science, Imam Mohammad Ibn Saud Islamic University (IMSIU), Riyadh 11623, Saudi Arabia; mejalouli@imamu.edu.sa; 3 Zoology Department, College of Science, King Saud University, Riyadh 11451, Saudi Arabia; hharrath@ksu.edu.sa; 4 Department of Pathology, College of Korean Medicine, Kyung Hee University, Seoul 02447, Republic of Korea; 2022310848@khu.ac.kr (J.C.); chlals2078@khu.ac.kr (M.C.); hyojeong25@khu.ac.kr (H.J.K.); shpark0912@khu.ac.kr (S.P.); eshimbs@khu.ac.kr (B.-S.S.); amama.rani@khu.ac.kr (A.R.); 5 Korean Medicine-Based Drug Repositioning Cancer Research Center, College of Korean Medicine, Kyung Hee University, Seoul 02447, Republic of Korea

**Keywords:** gastric cancer, nanotherapeutic delivery, drug resistance, tumor microenvironment, autophagy, targeted therapy

## Abstract

Gastric cancer (GC) is one of the most aggressive malignancies with a dismal prognosis, late diagnosis, and limited therapy efficacy. Biologically, GC is associated with multiple barriers to therapeutic response including gastric mucosal layer, acidic tumor microenvironment (TME), high accumulation of extracellular matrix (ECM) components, and limited penetration depth of anticancer drugs into tumor tissue. Furthermore, inherent or acquired drug resistance associated with drug efflux transporters, deregulated autophagy, tumor heterogeneity, and cell survival pathways severely compromise treatment response. Nanotechnology has been widely used to develop next-generation nanotherapeutic delivery systems to overcome these biological barriers. Currently available nanoplatforms such as liposomes, polymeric nanoparticles, dendrimers, and inorganic nanocarriers have improved drug loading capacity, aqueous solubility, circulation time stability, tumor-targeted delivery, and sustained release of chemotherapeutics. Smart and stimuli-responsive nanocarriers can also take advantage of pathological hallmarks of tumors including low pH, redox potential, and overexpressed enzymes for enhanced selective delivery to the tumor site. Nanotherapeutics have also shown promise for co-delivery of multiple therapeutic agents to overcome drug resistance, manipulation of TME, and suppression of autophagy and apoptosis signaling pathways associated with drug resistance. This review discusses recent advances in nanotherapeutics for GC including approaches to overcome biological barriers and drug resistance and highlights translational gaps for clinical development.

## 1. Introduction

Gastric cancer (GC) remains a leading cause of cancer-related mortality worldwide, characterized by late-stage diagnosis, high tumor heterogeneity, and limited therapeutic efficacy [1]. Although surgical resection in combination with chemotherapy, targeted therapy, and immunotherapy has improved survival rate to some extent, patients with advanced/metastatic GC still have a dismal prognosis [2]. Poor drug delivery of anti-GC therapeutic agents into tumor tissue due to numerous biological barriers remains one of the principal bottlenecks.

Biological barriers of GC mainly include gastric mucosal barrier, extreme acidity, dense extracellular matrix (ECM), tumor aberrant vasculature, which significantly limit drug stability, penetration, and accumulation [3]. In addition, the tumor microenvironment (TME) characterized by hypoxia, immunosuppression, and interaction with cancer-associated stroma promotes GC drug resistance [4]. GC cells can also acquire intrinsic or acquired drug resistance through multiple mechanisms, including upregulation of drug efflux pumps, activation of PI3K/AKT/mTOR and NF-κB pathways, inhibition of apoptosis and cell death, as well as autophagy activation [5]. Next-generation nanotherapeutic delivery systems based on nanotechnology have been proposed to overcome biological barriers and drug resistance for precise treatment of GC.

To date, a wide range of nanocarriers have been developed for gastric cancer treatment, including liposomes, polymeric nanoparticles, dendrimers, and inorganic nanoparticles [6]. They exhibit superiority in drug loading efficiency, prolonged systemic circulation, targeted delivery, sustained release, and real-time monitoring. In addition, smart or stimuli-responsive nanoplatforms could offer high tumor selectivity by responding to tumor microenvironmental cues such as pH, redox potential, enzymes, and biomolecules for GC therapy [7]. In this review, we summarized biological barriers and drug resistance mechanisms of GC and discussed the emerging advances of next-generation nanotherapeutic delivery systems for GC treatment.

## 2. Molecular Pathogenesis of Gastric Cancer

**Figure 1:**
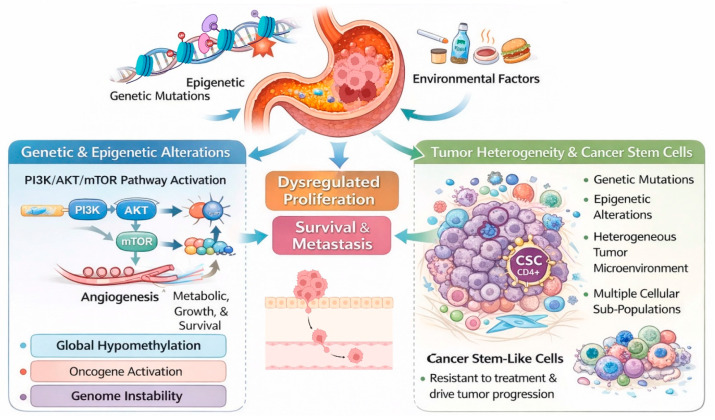
Molecular pathogenesis of gastric cancer. Genetic and epigenetic modifications, particularly the activation of the PI3K/AKT/mTOR signaling pathway, propel essential oncogenic processes such as enhanced cell proliferation, survival, angiogenesis, and metabolic reprogramming. Global DNA hypomethylation fosters genomic instability and activates oncogenes, hence advancing cancer. These molecular alterations cumulatively lead to dysregulated proliferation, increased survival, and metastatic advancement of gastric cancer cells. Tumor heterogeneity results from ongoing genetic and epigenetic changes within the tumor microenvironment, creating various cellular subpopulations with unique characteristics. Cancer stem-like cells (CSCs) are pivotal in tumor genesis, treatment resistance, and recurrence.

The development of gastric cancer is a multistep process that involves genetic mutations, epigenetic modifications, and environmental factors. Alterations in oncogenic signaling pathways and tumor suppressor genes lead to dysregulated proliferation, survival, and metastasis [8]. Tumor heterogeneity and cancer stem-like cells also play a role in gastric cancer progression (Figure 1). This interconnected network of molecular changes and cellular heterogeneity underlies gastric cancer growth and identifies critical targets for therapeutic intervention.

### 2.1. Genetic and Epigenetic Alterations

Genetic mutations commonly found in gastric cancer include loss of TP53 function. TP53 is involved in DNA repair, cell cycle arrest, and apoptosis [9]. The loss of TP53 function can lead to genomic instability that can lead to further genetic mutations. HER2/ERBB2 amplification or overexpression is found in gastric cancer and is used as a therapeutic target [10]. Targeting HER2 has been shown to improve outcomes in patients with HER2 overexpressing gastric cancers [11].

Activation of the PI3K/AKT/mTOR pathway is involved in gastric carcinogenesis. The activation of this pathway leads to increased cell growth, survival, proliferation, angiogenesis, and metabolic changes [12]. Activation of Wnt/β-catenin pathway has been shown to induce cellular proliferation, EMT, and gastric cancer invasion [13].

Epigenetic changes do not change the DNA sequence, but alter gene expression [14]. One epigenetic change is DNA methylation, which occurs through the addition of a methyl group to DNA [15]. Hypermethylation often occurs in tumor suppressor genes, which leads to transcriptional silencing of these genes. Global hypomethylation of the genome can also lead to carcinogenesis [16]. Histone modifications are changes to the histone proteins around which DNA winds. These modifications can include methylation, phosphorylation, and acetylation. Histone modifications can lead to alterations in the chromatin structure that can either promote or suppress the expression of genes involved in cancer [17].

### 2.2. Tumor Heterogeneity and Cancer Stem Cells

One hallmark of gastric cancer is tumor heterogeneity which contributes to treatment failure [18]. Intratumoral heterogeneity is the presence of subpopulations of cells with distinct genotypic and/or phenotypic profiles within a tumor, while intertumor heterogeneity is the variation seen between tumors from different individuals [19]. Tumor heterogeneity is dynamic and evolves during tumorigenesis due to genetic mutations, epigenetic alterations and the heterogeneous tumor microenvironment resulting in multiple cellular subpopulations within the same tumor [20]. This variation contributes to differences in cellular behaviors such as proliferation rate, metastatic capability and drug resistance which ultimately affects clinical outcome.

Gastric cancer stem-like cells (CSCs) represent a subpopulation of tumor cells with self-renewal, differentiation and tumor initiation capabilities and account for another level of tumor heterogeneity [21]. CSCs can evade therapy due to increased DNA repair capacity, upregulation of drug efflux transporters and activation of cell survival signaling pathways such as PI3K/AKT and Wnt/β-catenin, leading to recurrence and metastasis after treatment [22]. CSCs also interact with other components within the tumor microenvironment such as stromal cells, immune cells, and the extracellular matrix that maintain CSC properties. Tumor hypoxia and inflammatory cytokines that exist within the tumor niche also aid in CSC enrichment [23]. Therapeutic targets to overcome tumor heterogeneity will be discussed, with a focus on nanotherapeutics targeting gastric CSCs to prevent disease recurrence.

### 2.3. Linking Molecular Pathways to Nanotherapeutic Targeting Strategies in Gastric Cancer

The gap between molecular knowledge of tumor biology and successful therapeutic targeting is a significant obstacle in the treatment of gastric cancer. A logical technique to enhance treatment results is to combine important oncogenic pathways with nanotherapeutic delivery methods. The PI3K/AKT/mTOR signaling system, which is often active in stomach cancer, encourages cell survival, proliferation, and treatment resistance [24]. Delivery of pathway inhibitors or siRNA that targets AKT or PI3K via nanoparticles can improve intracellular drug accumulation and get around resistance mechanisms [25]. Similarly, cancer stem-like cells (CSCs), which are linked to drug resistance and tumor recurrence, are intimately linked to deregulation of the Wnt/β-catenin pathway [26]. CSC populations can be selectively suppressed by nanocarriers designed to deliver small-molecule inhibitors or gene-silencing drugs that target this pathway [27]. Additionally, autophagy-related pathways are essential for cytoprotective reactions to chemotherapy. Chemotherapeutic drugs and autophagy inhibitors, such as chloroquine, can be co-delivered by nanoparticles to interrupt autophagic flux and promote apoptosis [28,29]. Moreover, stimuli-responsive nanocarriers that release medications in low oxygen or acidic environments can be used to target tumor microenvironment-associated signaling, including hypoxia-driven pathways [30]. In general, precise targeting of tumor-specific mechanisms is made possible by connecting molecular pathways with nanotherapeutic design, providing a promising approach to overcoming biological barriers and drug resistance in gastric cancer.

## 3. Biological Barriers in Gastric Cancer Therapy

Delivery of therapeutic agents to gastric cancer is complicated by several biological barriers that impair drug stability, penetration and retention in tumor tissue. These barriers include the gastric mucosal layer, the acidic microenvironment, the dense extracellular matrix, and abnormal tumor vasculature. These barriers have limited effective treatment and lead to poor clinical responses and chemoresistance.

### 3.1. Gastric Mucosal Barrier and pH Constraints

**Figure 2:**
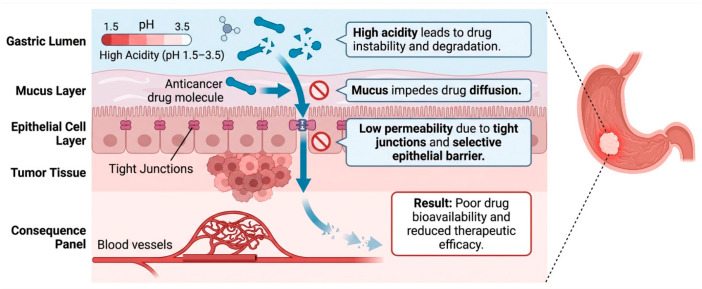
Biological barriers in gastric cancer therapy. Oral anticancer medications are unstable and degrade due to the gastric lumen’s extremely acidic environment (pH 1.5–3.5). The mucus layer beneath this serves as a physical barrier, limiting access to underlying tumor tissues and preventing medication diffusion. Because it is selective, the epithelial cell layer, which is strengthened by tight junctions, further limits drug penetration. Drug penetration into tumor tissue is severely hampered by these combined obstacles. As a result, poor bioavailability and decreased therapeutic efficacy are caused by limited medication absorption into the systemic circulation and tumor locations.

The natural protective barrier in the gastric region acts as the primary barrier for all the external insults, including orally administered drugs. Gastric mucosal barrier is highly selective due to its constituents like mucus, epithelial cells and tight junctions leading to poor drug permeability into the underlying tumor tissue [31]. Gastric lumen has high acidity with a pH ranging from 1.5 to 3.5 and the majority of drugs administered orally suffer instability [32]. Anticancer drugs especially chemotherapeutics are prone to acid-mediated degradation leading to lower bioavailability and therapeutic efficacy (Figure 2).

Acidic pH also affects ionization as well as solubility profile of the drugs hindering absorption and distribution of the drugs. Ionization of nanoparticle drug carriers could affect their stability and sustained release characteristics within the gastric region [33]. Hence designing nanoparticle drug carriers responsive to pH or protecting the drug within the carrier against acidic pH with specific targeting towards gastric tumor tissues is an important approach in gastric cancer treatment [34].

### 3.2. Tumor Microenvironment (TME)

**Figure 3:**
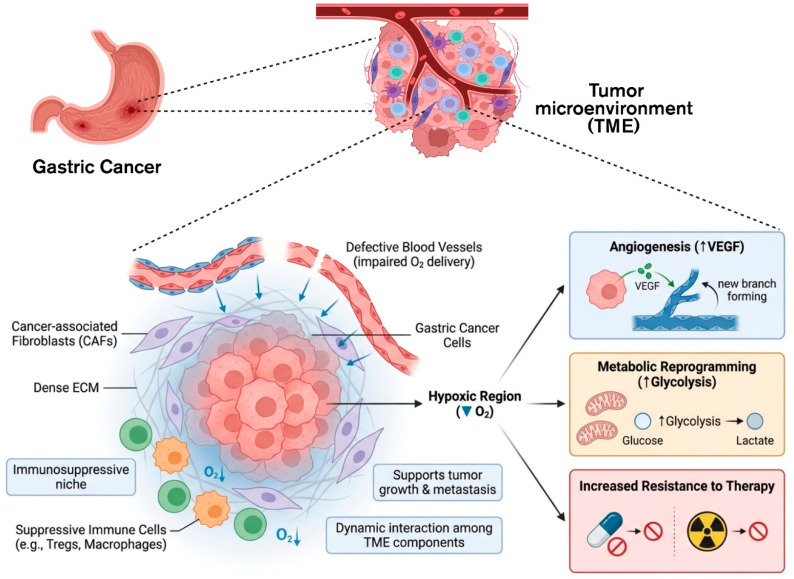
Biological barriers in tumor microenvironment. Cancer-associated fibroblasts (CAFs), dense extracellular matrix (ECM), immunosuppressive cells, and aberrant blood arteries make up the complex and dynamic microenvironment that characterizes gastric cancers. Hypoxic areas within the tumor are the result of poor oxygen delivery caused by defective vasculature. By upregulating vascular endothelial growth factor (VEGF) and causing metabolic reprogramming toward glycolysis, hypoxia increases angiogenesis and improves tumor survival. While immunosuppressive cells, such as regulatory T cells and tumor-associated macrophages, establish a suppressive niche that prevents anti-tumor immune responses, the extensive extracellular matrix and stromal components limit medication penetration. Tumor development, invasion, and metastasis are supported by interactions between these elements.

Gastric cancer tumor microenvironment (TME) consists of cellular and non-cellular components [35]. Cancer cells create an interactive niche that supports tumor growth and metastasis through cooperation with stromal cells, immune cells, and extracellular components. The tumor microenvironment is highly immunosuppressive and dynamic. A well-established hallmark of gastric cancer TME is hypoxia [36]. Tumor cells encounter low oxygen levels due to insufficient oxygen diffusion from defective vasculature. Hypoxia-inducible pathways in TME lead to angiogenesis, metabolic reprogramming, therapy resistance to chemotherapy, and radiotherapy (Figure 3). These elements work together to develop resistance to radiation and chemotherapy, underscoring the need to target the TME to enhance treatment efficacy in gastric cancer [37].

Several studies reported immune-suppressive pathways in gastric cancer TME. Regulatory T cells, tumor-associated macrophages, and inhibitory cytokines mediate immunosuppression leading to reduced anti-tumor immune activity in gastric cancer TME [38]. Furthermore, stromal pathways in gastric cancer lead to tumor progression. Cancer-associated fibroblasts and extracellular matrix (ECM) proteins promote tumor progression by augmenting cancer cell survival and restricting anti-tumor drug diffusion [39].

### 3.3. Extracellular Matrix (ECM) and Drug Penetration

The extracellular matrix (ECM) is a network of structural components in the tumor microenvironment that plays a significant role in drug delivery and response in gastric cancer [40]. The ECM is composed of collagen, fibronectin, hyaluronan, and other macromolecules that create a dense and highly organized scaffold around tumor cells [41]. The dense ECM can act as a physical barrier to drug delivery, limiting the penetration and distribution of therapeutic agents, including nanoparticles, into tumor tissues. High interstitial fluid pressure in tumors due to ECM accumulation and impaired lymphatic drainage can also hinder drug delivery. Cancer cell interactions with ECM components can also promote cell survival signaling, invasion, and metastasis [42]. Furthermore, ECM accumulation can physically impede the diffusion of oxygen and nutrients, leading to hypoxic regions that promote therapeutic resistance [43]. Strategies to overcome ECM-related barriers include enzyme-mediated degradation of the ECM, ECM-targeting ligands, and nanocarriers that can enhance tissue penetration.

### 3.4. Abnormal Tumor Vasculature

The tumor vasculature of gastric cancer exhibits abnormal morphology and function [44]. The tumor blood vessels are tortuous, leaky and poorly organized. As a result, the tumor blood vessels have heterogeneous blood flow causing poor drug delivery and heterogeneous distribution of drugs within the tumor tissue. Areas of low perfusion within the tumor result in hypoxic areas that drive tumor progression. The EPR effect allows nanoparticles to accumulate within tumor tissue [45]. However, poor vascularization within tumors leads to heterogeneous EPR effects. Lymphatic drainage is also impaired within tumors leading to high interstitial fluid pressure [46]. These factors limit drug delivery and distribution and should be taken into consideration when designing nanotherapeutics. Vascular normalization may be utilized to improve drug delivery as well as targeted delivery and stimulus-responsive nanocarriers.

### 3.5. Barriers to Clinical Translation

Although there are encouraging developments in the field of nanotherapeutics for gastric cancer, various challenges must be addressed before their widespread clinical translation. Scaling up production and ensuring reproducibility of complex nanocarriers with precise physicochemical properties can be technically demanding. The scalability of nanotherapeutic production processes and their associated high costs also present challenges [47]. Patient variability in response due to tumor heterogeneity and differences in the tumor microenvironment is another challenge. The regulatory approval process for nanomedicines can also be lengthy and complex [48]. Overcoming these challenges will require standardization of manufacturing processes, development of cost-effective production methods, and rigorous clinical validation.

## 4. Mechanisms of Drug Resistance in Gastric Cancer

**Figure 4:**
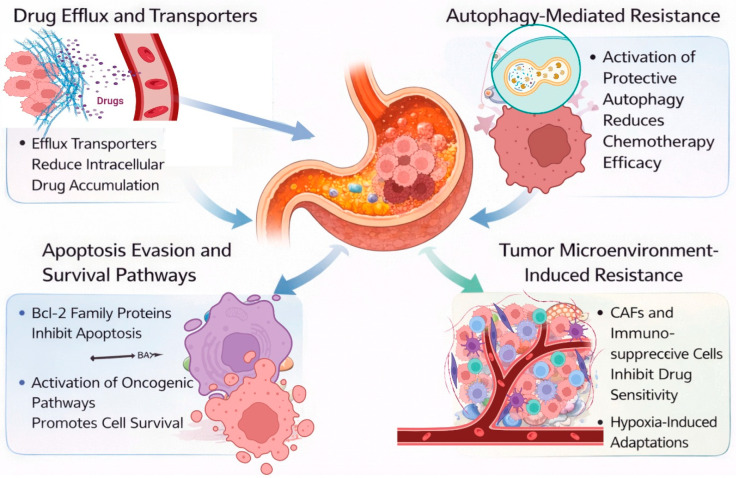
Mechanisms of drug resistance in gastric cancer. Drug efflux and transporter proteins, including ATP-binding cassette (ABC) transporters, actively diminish intracellular levels of chemotherapeutic drugs, thereby reducing their lethal effectiveness. Autophagy-mediated resistance allows cancer cells to endure therapeutic stress by activating protective autophagic pathways that mitigate chemotherapy-induced damage. Moreover, evasion of apoptosis and activation of survival signaling pathways, including the overexpression of anti-apoptotic proteins like Bcl-2 family members, enhance tumor cell survival and confer resistance to treatment. The tumor microenvironment exacerbates resistance via the presence of cancer-associated fibroblasts (CAFs), immunosuppressive cells, and hypoxia-induced adaptations, all of which diminish medication sensitivity and promote tumor growth.

Drug resistance is one of the greatest challenges in the treatment of gastric cancer. Multiple resistance mechanisms limit the clinical activity of both traditional chemotherapeutics and newer targeted agents. Mechanisms of drug resistance include drug efflux, autophagy, evasion of apoptosis, and alterations within the tumor microenvironment. Figure 4 shows a representation of the key mechanisms contributing to drug resistance in gastric cancer. These interrelated pathways collectively establish a formidable resistance network that diminishes the efficacy of standard treatments in gastric cancer.

### 4.1. Drug Efflux and Transporters

Overexpression of ATP-binding cassette (ABC) drug transporters, which actively pump out cytotoxic drugs, is another cause of drug resistance in gastric cancer cells [49]. ATP-dependent transporters such as P-glycoprotein (P-gp/ABCB1) and multidrug resistance-associated protein 1 (MRP1/ABCC1) limit intracellular accumulation of anticancer drugs and decrease their cytotoxic effects on cancer cells [49]. ABC drug transporters hydrolyze ATP to transport drugs including doxorubicin, paclitaxel, and cisplatin out of cells. Expression of these transporters can be upregulated by prolonged exposure to chemotherapeutic drugs and plays a major role in multidrug resistance. Inhibition of these transporters or evasion of transporter activity will be required to overcome therapeutic resistance.

### 4.2. Autophagy-Mediated Resistance

Autophagy can be both tumor-suppressive and tumor-promoting; however, in gastric cancer it has primarily been observed to promote tumor survival [50]. Autophagy induction acts as a cytoprotective mechanism when cancer cells experience stress from chemotherapy or other therapies [51]. In addition to chemotherapy stress, autophagy induction has been shown under hypoxic conditions and nutrient deprivation in gastric cancer cells [52]. Autophagy allows cancer cells to recycle intracellular material to provide energy and other necessary components during times of stress. LC3 (Microtubule-associated protein 1 light chain 3) and p62/SQSTM1 are autophagy related proteins that regulate autophagy induction [53].

LC3 turnover from LC3-I to LC3-II and p62 degradation is measured to monitor autophagy flux [54]. Expression of LC3 and depletion of p62 have been shown to increase autophagy flux and induced resistance to anticancer therapies [55]. Induction of autophagy allows cancer cells to prevent apoptosis during times of stress from chemotherapeutic agents [56]. Autophagy is also highly regulated by cell signaling pathways including PI3K/AKT/mTOR and AMPK. Autophagy inhibition by chemical compounds has been shown to decrease chemoresistance in gastric cancer [57]. Therefore, autophagy can be targeted to reduce drug resistance in gastric cancer [58].

### 4.3. Apoptosis Evasion and Survival Pathways

Evading apoptosis, another cancer hallmark, has been found to participate in therapeutic resistance [59,60]. This resistance develops as cancer cells gain the ability to escape cell death through the dysregulation of pathways promoting or inhibiting apoptosis [29]. The PI3K/AKT/mTOR pathway signaling leads to tumor cell survival, proliferation, and metabolism [25]. The pathway has been shown to inhibit apoptosis through suppressing the expression of pro-apoptotic proteins such as Bax and increasing the expression of anti-apoptotic proteins such as Bcl-2 [61,62].

NF-κB signaling is also commonly activated in gastric cancer and is known to promote inflammation-associated cancer initiation, development, and therapeutic resistance [63]. NF-κB can induce cell survival and proliferation genes, inhibit apoptosis, and is activated by various stress signals including chemotherapy [64]. Mutations or alterations in TP53 disrupt apoptotic cell death signaling pathways in response to DNA damage.

### 4.4. Tumor Microenvironment-Induced Resistance

The tumor microenvironment (TME) can also promote drug resistance in gastric cancer [38]. Hypoxia within the TME leads to the activation of hypoxia-inducible factors (HIFs), which contribute to angiogenesis, metabolic reprogramming, and resistance to chemotherapy and radiotherapy [65]. Hypoxia also promotes autophagy and decreases drug-induced apoptosis [66].

Moreover, immune evasion in the TME, including regulatory T cells and tumor-associated macrophages, inhibits anti-tumor immune responses [67]. These mechanisms work together to create a protective environment for cancer cells against drugs. Targeting mechanisms associated with TME may help overcome drug resistance. Other mechanisms of apoptosis evasion include alterations in crosstalk between these pathways. Targeting cell survival pathways through inhibition or combination therapy may overcome therapeutic resistance by re-sensitizing cells to apoptosis [68].

## 5. Next-Generation Nanotherapeutic Delivery Systems

**Figure 5:**
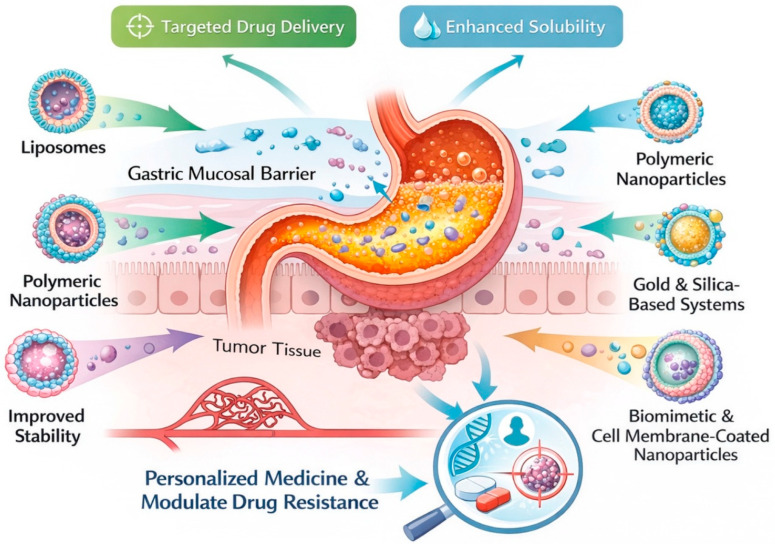
Next-generation nanotherapeutic delivery systems. The stomach mucosal barrier can be overcome by a variety of nanocarriers, including liposomes, polymeric nanoparticles, gold and silica-based nanoparticles, and biomimetic cell membrane-coated nanoparticles. These technologies facilitate targeted delivery to tumor sites while enhancing medication solubility, stability, and circulation. Nanoparticles improve accumulation within tumor locations, enable controlled and prolonged medication release, and permit effective transport across biological barriers. By increasing selectivity for cancer cells, functionalization with targeted ligands reduces off-target effects. Biomimetic nanoparticles also offer extended systemic circulation and immune evasion.

Advanced nanotherapeutic delivery systems have been developed as effective modalities to enhance drug delivery for gastric cancer. By targeting drug barriers and improving solubility, stability, targeting, and controlled release, nanoparticle platforms can successfully deliver drugs across biological barriers and increase their potency. Various nanoparticles provide distinct benefits and can be used for personalized medicine or to modulate drug resistance (Figure 5). Additionally, Table 1 shows that liposomal and polymeric nanocarriers exhibit the greatest translational potential, while biomimetic and inorganic nanoparticles present novel yet nascent approaches to surmount biological barriers and drug resistance in gastric cancer.

### 5.1. Liposomes and Polymeric Nanoparticles

Liposomes and polymeric nanoparticles are two of the most investigated nanocarriers for gastric cancer therapy. Liposomes are phospholipid-based vesicles that can encapsulate both hydrophilic and hydrophobic drugs, improving their solubility and stability. They are biocompatible and resemble biological membranes, which allows for easy cellular uptake and reduced toxicity. Modification with polyethylene glycol (PEG) can increase circulation time by avoiding opsonization and clearance by the reticuloendothelial system.

Polymeric nanoparticles are made of biodegradable polymers such as PLGA and chitosan. They can provide controlled and sustained release of drugs and can be designed to respond to stimuli present in the tumor microenvironment, such as pH or enzymes, for site-specific drug delivery. Furthermore, they can be functionalized with targeting ligands, such as antibodies or peptides, for enhanced tumor targeting. Overall, liposomes and polymeric nanoparticles have shown great promise in improving pharmacokinetics, drug accumulation, and therapeutic efficacy in gastric cancer treatment.

### 5.2. Dendrimers and Micelles

Dendrimers and polymeric micelles are nanocarriers with precisely defined structures. They can also achieve high loading of drugs within their structures. Dendrimers are nanosized macromolecules characterized by branching tree-like structures with numerous terminal functional groups. Drugs can be conjugated to these surface groups with precision, enabling targeting at the nanoscale. Like liposomes, dendrimers can also be modified with targeting ligands or imaging agents to produce theranostic agents.

Polymeric micelles are nanocarriers formed by the self-assembly of amphiphilic block copolymers. The interior of these micelles is hydrophobic, allowing poorly soluble anticancer drugs to be loaded. The outer shell is hydrophilic, allowing the micelle to be stable while traveling in the circulatory system. Micelles can be engineered to respond to changes in the environment to release their payload at the desired site.

Dendrimers and micelles are useful nanocarriers that can increase the bioavailability of drugs, minimize toxicity and help target drugs more effectively against gastric cancer.

### 5.3. Inorganic Nanoparticles

Metal nanoparticles such as gold nanoparticles have unique physicochemical properties and multifunctional features such as superior biocompatibility, facile surface modification, and photothermal capabilities for combination therapy applications. Silica-based nanocarriers offer advantages like high surface area and tunable pore structure for efficient drug loading. Furthermore, they can be easily modified with tumor-targeting ligands to enhance tumor targeting and cellular internalization. The imaging capabilities of inorganic nanoparticles also allow theranostic applications. In gastric cancer, inorganic nanoparticles allow for numerous strategies to be applied for therapy.

### 5.4. Biomimetic and Cell Membrane-Coated Nanoparticles

Biomimetic nanoparticles and cell membrane-coated nanoparticles are another class of nanoparticles that aid immune evasion and tumor targeting. Membranes from red blood cells, cancer cells, and immune cells have been used to cloak nanoparticles to increase their circulation time in the blood flow and prevent clearance from the body. They maintain homotypic targeting ability and ligands on the membranes that can promote interactions with tumor cells. Biomimetic nanoparticles have also been shown to have effects on tumor microenvironments as well as improving drug delivery to tumors.

## 6. Smart and Stimuli-Responsive Nanotherapeutics

Smart nanotherapeutics are designed to respond to stimuli associated with tumors for controlled drug release specifically in gastric cancer. pH-, redox-, and enzyme-responsive drug delivery take advantage of tumor microenvironment differences and allow for targeted treatment with minimal side effects.

### 6.1. pH-Responsive Systems

Drug delivery systems based on pH-sensitive materials respond to extracellular acidic environment as well as to intracellular compartments, including endosomes and lysosomes. pH-sensitive drug delivery systems are stable at physiological pH but change conformation or degrade in acidic conditions, inducing drug release. These materials include pH-sensitive polymers and acid-labile linkers for site-specific drug delivery, preventing premature leakage. pH-sensitive nanocarriers take advantage of the acidic nature of gastric tumor microenvironment and gastric lumen. pH-responsive nanoparticles applied for the treatment of gastric cancer led to improved stability and increased accumulation of the drug in the tumor region leading to better efficacy with reduced side effects.

### 6.2. Redox-Responsive Nanocarriers

Redox-responsive nanocarriers can take advantage of the elevated levels of intracellular glutathione (GSH) present in many cancer cells. Nanocarriers can be synthesized with redox-sensitive bonds (i.e., disulfide linkages) that are stable outside the cell but cleaved under reductive conditions. Upon entering tumor cells, disulfide bonds are cleaved by high concentrations of GSH, leading to fast release of drug. This type of controlled release mechanism allows for increased intracellular concentrations of the drug while decreasing total systemic exposure. Gastric cancer presents an opportunity for redox-responsive nanocarriers to increase therapeutic efficacy by targeting drugs to tumor cells.

### 6.3. Enzyme-Responsive Platforms

Tumor-specific enzymes overexpressed in gastric cancer like matrix metalloproteinases (MMPs), cathepsins and other proteases involved in tumor progression and extracellular matrix remodeling have been exploited to design enzyme-responsive nanotherapeutic systems. Enzyme-cleavable linkers can be incorporated within nanocarriers that are stable in normal tissues but readily cleaved upon exposure to target enzymes for site-specific drug release with higher therapeutic efficacy and lower off-target toxicity. In addition, enzyme responsive platforms have been proposed to enable better tumor penetration through degradation of the extracellular barriers.

## 7. Nanotherapeutic Strategies to Overcome Drug Resistance

**Figure 6:**
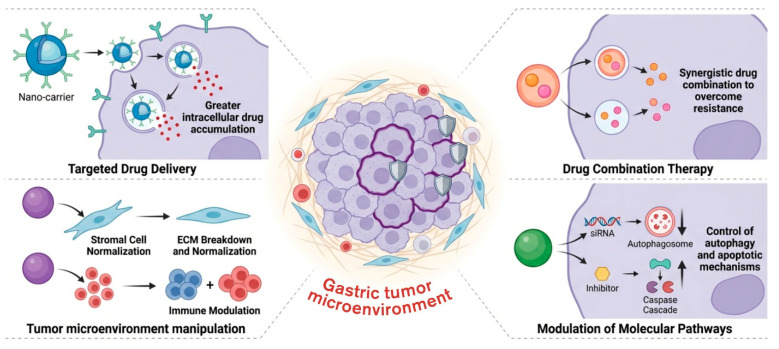
Nanotherapeutic strategies to overcome drug resistance. Enhancing intracellular drug accumulation through targeted drug delivery with functionalized nanocarriers improves therapeutic efficacy. Co-delivery technologies allow for the suppression of resistance-associated pathways by synergistic drug combination therapy, such as chemotherapy with siRNA or miRNA. By controlling autophagy and apoptosis, nanotherapeutics also alter important molecular processes, such as the activation of caspase-mediated cell death pathways and the suppression of autophagosome formation. Furthermore, by encouraging stromal normalization, extracellular matrix (ECM) breakdown, and immunological modulation, nanoparticles might alter the tumor microenvironment.

Nanotherapeutics offer a unique opportunity to circumvent gastric cancer drug resistance. Targeted drug delivery, drug combination therapy, and inhibition or activation of molecular targets associated with drug resistance are just some of the functions nanotherapeutic systems can possess. The use of nanoparticle delivery techniques to address multidrug resistance in gastric cancers are examined. This compilation of recent advancements in this domain can enhance comprehension of novel methodologies [69]. These systems allow for greater intracellular accumulation of chemotherapeutics, control of autophagy and apoptotic mechanisms, and manipulation of the gastric tumor microenvironment (Figure 6).

### 7.1. Co-Delivery Systems

Co-delivery nanotherapeutics have shown great promise in tackling drug resistance in gastric cancer. By combining chemotherapeutic drugs with gene-regulating molecules such as small interfering RNA (siRNA) or microRNA (miRNA), these platforms allow for the simultaneous delivery of multiple therapeutic agents to tumor cells. Co-delivery systems can provide synergistic therapeutic effects and reduce the likelihood of resistance development.

Examples include nanoparticles co-loaded with doxorubicin or paclitaxel and siRNA targeting ABC transporters (e.g., P-gp) or anti-apoptotic proteins (e.g., Bcl-2). Nanoparticles can deliver both the chemotherapeutic drug and siRNA into tumor cells, leading to increased intracellular drug accumulation, inhibition of drug efflux pumps, and induction of apoptosis. Similarly, co-delivery of miRNAs that target oncogenic pathways and restore tumor suppressor functions can be achieved.

Common nanocarriers used for co-delivery include liposomes, polymeric nanoparticles, and dendrimers. These carriers can be modified with targeting ligands on their surface to enhance tumor targeting and cellular uptake. Nanocarriers can also provide protection for the nucleic acids from degradation and have high loading capacity for both drugs and nucleic acids. Overall, co-delivery nanotherapeutics offer a versatile approach to synergistically target multiple pathways involved in drug resistance and improve therapeutic outcomes in gastric cancer.

### 7.2. Targeting Autophagy and Apoptosis

Autophagy and apoptosis are also exploited by cancer cells to develop resistance to chemotherapy. One way in which cancer cells develop resistance is by using cytoprotective autophagy during times of stress, such as chemotherapy treatment. Nanotherapeutics that either suppress cytoprotective autophagy or induce excessive autophagy have potential as therapeutic agents to combat chemotherapy resistance.

Nanotherapeutics could combine autophagy inhibitors like chloroquine or hydroxychloroquine with chemotherapeutics. These autophagy inhibitors act by suppressing autophagosome-lysosome fusion and blocking autophagic flux. Nanocarriers provide advantages such as co-delivery, synchronized targeting, and enhanced intracellular uptake compared to free drug delivery. When autophagy is inhibited using nanoparticles co-delivering chloroquine or hydroxychloroquine and chemotherapy drugs, cancer cells accumulate LC3-II and p62 and are sensitized to chemotherapy. Interestingly, this sensitization to chemotherapy induced by autophagy inhibition has also been shown to occur through induction of endoplasmic reticulum stress.

Autophagy can also be targeted to induce excessive autophagy leading to cell death. Nanotherapeutics designed to target apoptosis signaling pathways could also be beneficial for treating cancer. Targeting components of the apoptosis pathway like Bcl-2 family proteins, caspases, and mitochondrial components could help reactivate apoptotic cell death in tumor cells. By combining both autophagy suppressors or enhancers with apoptotic inductors, a single nanoparticle could potentially overcome resistance and effectively treat cancer.

### 7.3. Modulation of Tumor Microenvironment

Targeting the tumor microenvironment (TME) is another potential strategy for overcoming drug resistance in gastric cancer. Gastric cancer is characterized by various microenvironmental features such as hypoxia, immune suppression, and stromal interactions. Nanotherapeutic systems can be developed to specifically target and reprogram these microenvironmental characteristics.

Nanoparticles can be engineered to target hypoxic regions within tumors. Hypoxia-targeting nanoparticles can selectively activate or release drugs in response to low oxygen levels, improving treatment response and reducing resistance. Nanocarriers can also deliver hypoxia-sensitive prodrugs or oxygen-generating molecules to improve treatment efficacy and overcome resistance.

Targeting immunosuppressive components of the TME can also enhance treatment response. Nanoparticles can be loaded with immunotherapeutic agents, cytokines, or immune checkpoint inhibitors to modulate the immune response and promote anti-tumor immunity. Nanotherapeutic systems can also target stromal components within the TME, such as cancer-associated fibroblasts and extracellular matrix, to improve drug penetration and distribution within tumors. By modulating microenvironmental factors associated with drug resistance, nanotherapeutics can improve drug delivery, overcome resistance mechanisms, and enhance treatment efficacy in gastric cancer.

## 8. Translational and Preclinical Perspectives of Nanotherapeutics in Gastric Cancer

Nanotherapeutic approaches for gastric cancer have shown considerable potential in preclinical models, especially in enhancing drug delivery, surmounting biological obstacles, and mitigating drug resistance. In vitro and in vivo investigations utilizing gastric cancer cell lines and xenograft models have demonstrated that nanocarriers, including liposomes, polymeric nanoparticles, micelles, augment drug accumulation, extend circulation duration, and boost treatment efficacy relative to free medicines [70]. These technologies provide the simultaneous administration of chemotherapeutics alongside siRNA or miRNA to inhibit resistance-associated pathways, such as ABC transporters and autophagy-related signaling [71]. Notwithstanding these promising results, their use in clinical practice is still constrained. A limited number of nanotherapeutic platforms, including liposomal doxorubicin and nanoparticle albumin-bound paclitaxel (nab-paclitaxel), have progressed to clinical assessment, exhibiting enhanced safety profiles and moderate therapeutic advantages in patients with gastric cancer [72]. Nevertheless, numerous next-generation nanocarriers, such as biomimetic nanoparticles and stimuli-responsive systems, are still in the preclinical phase due to difficulties in scalability, repeatability, and regulatory approval [73,74]. Connecting preclinical success to clinical application necessitates consistent evaluation techniques, comprehensive pharmacokinetic and toxicity research, and well-organized clinical trials [75]. The ongoing integration of molecular targeting methods and tailored approaches will be crucial for expediting the clinical translation of nanotherapeutics in gastric cancer. Table 2 represents nanotherapeutic platforms in gastric cancer currently used in preclinical and clinical studies.

## 9. Translational and Clinical Perspectives

To date, the clinical translation of nanotherapeutic modalities for gastric cancer has remained limited, despite highly encouraging preclinical results. Some improvements regarding efficacy, safety, scalability, or regulatory approval issues need to be solved. Recognizing clinical advances and translational barriers will help propel next-generation nanomedicine for gastric cancer into standard-of-care therapies.

### 9.1. Preclinical and Clinical Progress

Major advancements have been achieved over the years in preclinical translation of nanotherapeutic platforms for gastric cancer. Numerous nanotherapeutic investigations have shown promising preclinical outcomes in gastric cancer, encompassing increased drug delivery, augmented tumor-targeting efficacy, and diminished systemic toxicity. Drugs, when encapsulated into nanocarriers such as liposomal formulations, polymeric nanoparticles, and micelles, show improved PK profile and therapeutic efficacy.

Multiple nanomedicine-based drug formulations like liposomal doxorubicin and nanoparticle albumin-bound paclitaxel (nab-paclitaxel) have entered clinical trials with improved response rates and lower toxicity profiles as compared to conventional chemotherapy. Targeted nanocarriers and drug combinations aiming at overcoming therapeutic resistance are also undergoing clinical trials. Recent developments in imaging-guided nanomedicine platforms and theranostic nanoplatforms are also contributing to personalized medicine approaches. Development of nanotherapeutics in clinical settings is still underway but shows great potential.

### 9.2. Safety, Toxicity, and Regulatory Challenges

Safety and toxicity are important issues that need to be considered when implementing nanotherapeutics clinically. Nanocarriers are generally designed to be biocompatible and biodegradable; however, their long-term safety effects still need to be evaluated, and potential clearance failures may cause accumulation of carriers in the liver and spleen. Immunogenicity, off-target toxicities, and nanoparticle toxicity should be addressed during preclinical and clinical studies.

A challenge facing nanomedicine regulations is characterizing the often-complex composition of nanomaterials that vary widely in size, composition, surface charge, and chemistry. Standard characterization protocols as well as safety testing and quality control need to be developed and implemented to allow safe and uniform clinical translation of nanotherapeutics.

## 10. Key Translational Gaps and Conceptual Framework for Future Research

Even while nanotherapeutic delivery technologies for stomach cancer have advanced significantly, there are still several important gaps that prevent successful clinical translation. The tumor microenvironment’s heterogeneity, which causes uneven medication accumulation and varied therapy results, is one significant drawback. The reliance on the enhanced permeability and retention (EPR) effect remains problematic, as its efficiency differs across patients and tumor types [83]. Furthermore, many nanocarriers’ efficacy is limited by inadequate tumor penetration, especially when there is a strong extracellular matrix and high interstitial pressure [84]. Clinical development is further complicated by safety issues like immunogenicity, long-term toxicity, and off-target accumulation. Furthermore, issues with reproducibility, scalable production, and regulatory uniformity continue to be major obstacles.

We propose a conceptual paradigm for next-generation nanotherapeutic design in gastric cancer to overcome these constraints. This framework incorporates three essential elements: mechanism-driven targeting, which addresses drug resistance pathways like autophagy, apoptosis, and efflux transporters; barrier-oriented design, which focuses on overcoming physiological and microenvironmental constraints like pH, hypoxia, and ECM density; and precision-guided delivery, which uses patient-specific molecular profiling and AI-assisted optimization [85]. This integrated approach provides a rational roadmap for developing more effective and translatable nanotherapeutic strategies for gastric cancer.

Future directions in the development of nanotherapeutic delivery for gastric cancer involve precision nanomedicine and personalized therapy approaches. Tailoring nanocarriers based on patient-specific molecular profiling (genomic, transcriptomic, and proteomic data) can enhance therapeutic targeting and reduce off-target effects. Personalized nanomedicine can allow for selective targeting of relevant pathways involved in tumor growth, drug resistance, and metastasis. The combination of nanotherapeutics with immunotherapy holds promise in gastric cancer treatment. Nanocarriers can be engineered to deliver immune checkpoint inhibitors, cytokines, or tumor-associated antigens to stimulate anti-tumor immune responses and overcome immune suppression in the tumor microenvironment. Such combination therapies may lead to more durable responses. Additionally, nanogels offer distinctive advantages for the stabilization of delicate enzymes via pliable, hydrated polymer networks [86]. Artificial intelligence (AI) may also play a role in the development of nanotherapeutics. AI algorithms can be used to design nanoparticles with optimized composition and predict drug release kinetics and delivery efficiency. AI-assisted models can also help identify potential combination therapies. These advancements may facilitate the development of next-generation nanomedicines for gastric cancer and enhance their translation to the clinic.

## 11. Conclusions

Next-generation nanotherapeutic delivery platforms hold great promise in overcoming challenges such as biological barriers and drug resistance associated with gastric cancer treatment. These platforms can improve drug stability, targeting ability, and controlled release profiles, thereby enhancing therapeutic efficacy. Recent advancements in intelligent, stimuli-responsive nanocarriers and combinational strategies targeting autophagy, apoptosis, and the tumor microenvironment further bolster the clinical potential of nanotherapeutics in gastric cancer. However, challenges related to safety, scalability, and regulatory hurdles need to be addressed for successful translation. The future integration of nanotechnology with precision medicine, immunotherapy, and artificial intelligence holds promise in advancing effective and personalized treatment modalities for gastric cancer.

## Figures and Tables

**Table 1:** Comparative analysis of next-generation nanotherapeutic delivery systems in gastric cancer.

Nanoplatform	Key Features	Advantages	Limitations	Targeting Strategy	Translational Potential	Relevance to Gastric Cancer
Liposomes	Phospholipid vesicles encapsulating hydrophilic and hydrophobic drugs	High biocompatibility, improved solubility and stability, reduced toxicity, PEGylation enhances circulation time	Limited tumor penetration, drug leakage, dependence on EPR effect	Passive (EPR), PEGylation, ligand modification	High (clinical use available)	Effective for improving pharmacokinetics and reducing systemic toxicity
Polymeric Nanoparticles (PLGA, chitosan)	Biodegradable polymer-based carriers with controlled release properties	Sustained drug release, high stability, tunable design, stimuli-responsive delivery	Scale-up challenges, batch variability, possible polymer toxicity	Passive + Active (ligands, antibodies)	Moderate–High (some clinical translation)	Suitable for overcoming acidic TME and enabling targeted delivery
Dendrimers	Highly branched, tree-like macromolecules with multiple functional groups	Precise drug conjugation, high loading capacity, multifunctional (targeting + imaging)	Complex synthesis, potential cytotoxicity, clearance issues	Active targeting via surface functionalization	Low–Moderate (mostly preclinical)	Useful for gene delivery and combination therapy
Polymeric Micelles	Self-assembled amphiphilic structures with hydrophobic core	Ideal for poorly soluble drugs, improved stability in circulation, controlled release	Instability upon dilution, premature drug release	Passive targeting, stimuli-responsive	Moderate (limited clinical use)	Enhances delivery of hydrophobic chemotherapeutics
Inorganic Nanoparticles (Gold, Silica)	Metal or silica-based nanostructures with tunable physicochemical properties	High surface area, imaging capability (theranostics), photothermal effects	Long-term toxicity, poor biodegradability, clearance concerns	Active + Stimuli-responsive	Low (preclinical stage)	Useful for combination therapies (e.g., photothermal + chemotherapy)
Biomimetic/Cell Membrane-Coated Nanoparticles	Nanoparticles coated with cell-derived membranes (RBCs, cancer cells, immune cells)	Immune evasion, prolonged circulation, homotypic targeting, enhanced tumor interaction	Complex fabrication, reproducibility issues, scalability challenges	Biomimetic targeting (natural ligands)	Emerging (early preclinical)	Promising for TME modulation and overcoming immune-related barriers

**Table 2:** Representative nanotherapeutic platforms in gastric cancer.

Nanoplatform	Payload	Targeting Strategy	Development Stage	Key Limitations	Ref.
Liposomes (e.g., Liposomal Doxorubicin)	Doxorubicin	Passive (EPR), PEGylation	Clinical	Limited tumor penetration, EPR variability	[76]
Polymeric Nanoparticles (PLGA, chitosan)	Paclitaxel, Cisplatin	Passive + Active (ligands)	Preclinical/Clinical	Scale-up challenges, batch variability	[77]
Micelles	Hydrophobic drugs (e.g., docetaxel)	Passive targeting	Clinical/Preclinical	Stability issues in circulation	[78]
Dendrimers	Drugs, siRNA	Active targeting (surface functionalization)	Preclinical	Toxicity concerns, complex synthesis	[79]
Inorganic Nanoparticles (Gold, Silica)	Drugs, photothermal agents	Active + Stimuli-responsive	Preclinical	Long-term toxicity, clearance issues	[80]
Biomimetic Nanoparticles	Drugs, nucleic acids	Cell membrane-mediated targeting	Preclinical	Manufacturing complexity, reproducibility	[81]
Nab-paclitaxel	Paclitaxel	Albumin-mediated targeting	Clinical	Cost, limited targeting specificity	[82]

## Data Availability

No new data were created or analyzed in this study.
